# Data on transgenerational memory effects of photosynthetic efficiency of twelve wheat varieties under elevated carbon dioxide concentration and reduced soil water availability

**DOI:** 10.1016/j.dib.2025.111545

**Published:** 2025-04-09

**Authors:** Bernd J. Berauer, Suraj Chaudhary, Lorenz Kottmann, Andreas H. Schweiger

**Affiliations:** a Department of Plant Ecology, Institute of Landscape and Plant Ecology, University of Hohenheim, Stuttgart, Germany; b Julius Kühn-Institute (JKI) – Federal Research Centre for Cultivated Plants, Institute for Crop and Soil Science, Braunschweig, Germany

**Keywords:** Photosynthesis, Wheat, Epigenetic, Ecophysiology, Climate change, ACi curve, Carboxylation rate, Electron transport

## Abstract

This data [1] represents ACi curves of twelve winter wheat varieties, which were grown under elevated and ambient CO_2_ concentrations within a FACE experiment and the subsequent F1 generation was exposed to ambient and elevated CO_2_ concentrations in a highly controlled environment using climate chambers. The 12 winter wheat genotypes (*Triticum aestivum* L.) were selected based on their susceptibilty to leaf rust (*Puccinia triticina* Eriks.) and Fusarium head blight (*Fusarium graminearum* Schwabe) according to the descriptive variety list of the German Federal Office of Plant Varietes (Beschreibende Sortenliste, Bundessortenamt 2024). The aim was to obtain a diverse set of varieties with the widest possible range of susceptibilities to leaf rust and fusarium head blight. Photosynthesis was measured using the novel Dynamic Assimilation Technique, thus not with the common steady-state approach. The individual wheat plants were measured twice, once under saturating soil water availability (θ_FC_) and once under reduced soil water availability (θ_csoil_). θ_csoil_ represents the gravimetric water content when the soil matric potential drops below the root matric potential, thus the onset of plant drought stress (*sensu* Cai et al*.* [2]). The photosynthesis data was used to fit ACi curves and extract the maximum Rubisco carboxylation rate [Vc_max_], maximum rate of electron transport [J_max_] and dark respiration [Rd]. At both measurements we determined BBCH and plant height to quantify plant morphological development, as well as leaf water potential to quantify plant ecohydrologic status. At the end of the experiment, biomass was harvested and reported. Further, we provide environmental data of the climate chambers in use.

Within the data repository, we provide comprehensive experimental data on the investigation of transgenerational memory effects on photosynthetic efficiency. We provide photosynthetic raw data as well as processed (merged) and derived (extracted ACi fit) data. Additionally, we provide the R-code to reproduce the calculation of the derived parameters.

Data on transgenerational memory effects (that is, the influence of the parental environment on offspring phenotype and performance) are scarce, i.e. on the adaptive capacity of the photosynthetic apparatus. Thus, the data provided here can contribute to closing this gap. The highly controlled environment allows to closely investigate cause-effect relationships, thereby contributing to a mechanistic understanding of the transgenerational memory effects on photosynthetic efficiency and how this is altered by reduced soil water availability. By using a recently developed methodological approach, the data contributes to further investigate the quality of the method and establish it within the field of plant ecophysiology.

Specifications Table

**Table:** 

Subject	Biology
Specific subject area	*Plant Ecophysiology: adaptation of photosynthetic efficiency to increased carbon dioxide across multiple generations of agronomic-used wheat varieties*
Type of data	Raw. Processed data. Additional derived parameters using up-to-date computational methodology.
Data collection	Twelve agronomic winter wheat varieties were grown under highly controlled environmental conditions in climate chambers. These varieties were grown under two carbon dioxide concentrations at their maternal and the F1 generation. We measured photosynthesis under saturating and reduced soil water. Photosynthesis data was collected using two Li-6800 (LiCor, Lincoln, Nebraska, USA) following the recently developed Dynamic Assimilation Technique. Leaf water potential was measured using a scholander pressure bomb (Model 1000, PMS Instrument Company, USA). Experimental data such as climate chamber environmental conditions, plant irrigation amount and biomass at harvest are documented.
Data source location	Country: Germany Institution: University of Hohenheim – Department of Plant Ecology*.*
Data accessibility	Repository name: Zenodo Data identification number: 10.5281/zenodo.15057328 Direct URL to data: https://zenodo.org/records/15185206 Instructions for accessing these data: None
Related research article	None

## 1 Value of the Data

• The presented data set quantifies transgenerational memory effects (i.e. the influence of the parental environment on offspring phenotype and performance) of photosynthetic efficiency to increased carbon dioxide conditions and how these effects are altered by reduced soil water availability. To quantify the photosynthetic response we applied a novel methodological approach (Dynamic Assimilation Technique instead of classic steady-state measurements) under highly controlled environmental conditions.
• This dataset can contribute to quantify epigenetic/transgenerational effects on the photosystem of plants. By this, it can contribute to understand and improve the adaptive capacity of wheat plants under future climatic scenarios.
• Providing further data using a novel methodology will help to investigate the method's robustness and increase its visibility and propagate its advantages.
• Data on transgenerational memory effects (i.e. the influence of the parental environment on offspring phenotype and performance) are scarce, i.e. on the adaptive capacity of the photosynthetic apparatus. Thus, the data provided here can contribute to closing this gap.
• The data allows to quantify the effect of two major global change impacts, namely increasing atmospheric carbon dioxide concentrations and reduced soil water availability on plants' ecophysiologic performance and on the transgenerational memory.

## 2 Background

Increasing atmospheric CO_2_ and drought are major symptoms of anthropogenic climate change [3] with profound effects on plant growth, ecosystem functioning and agronomic yields [4]. Transgenerational memory (i.e. the influence of the parental environment on offspring phenotype and performance) has been suggested as a relevant mechanism for plants to build up adaptative capacity for rapid environmental changes [5]. However, this mechanism of pre-adaptation remains poorly investigated so far.

Photosynthesis is the key process of carbon acquisition. Plant carbon uptake is regulated by stomatal opening, so is transpirational water loss. Thus, mechanisms of plants to prevent drought stress, such as stomata closure, directly interfere with photosynthetic carbon acquisition [6]. Higher atmospheric CO_2_ concentrations increase photosynthetic carbon acquisition by increasing intercellular CO_2_ concentrations and carboxylation efficiency as well as by reducing photorespiration [7]. By this increasing atmospheric CO_2_ concentrations can reduce photosynthesis limitation by reduced soil water availability.

Given the pace of current environmental change, it is important to investigate the potential of transgenerational memory, which remains longer active than acclimation and enables plants to respond faster and more adequately to subsequent stress events [8].

## 3 Data Description

The repository is structured hierarchically using folders.

The ***R_code*** folder contains one PDF containing the commented R-code to be able to process the raw data and calculate the fitted Aci-curves as well as the R-markdown file, which can directly be loaded into the respective software. Further, it contains the session information used to execute the code, which might be important for future use as package dependencies might change.

The ***Data*** folder contains one folder and one Excel-workbook. Within the folder ***photosynthesis_raw*** the unprocessed raw data - directly exported from the portable photosynthesis instruments - is stored. This ***photosynthesis_raw*** folder is further substructured in two folders (one for each of the two identical Li-6800 instruments used). Within those the raw data is stored in folders for the respective measurement date. The raw data contains each file name twice, once with the LiCor-specific file extension and once with .xlsx file extension (as this reflects the *default* setting of data export from the Li-6800). The naming of the files follow a common theme including the date and time of the file creation, the climate chamber (ch2, ch3, ch4, ch6) and the replicate ID (PotNr) resulting in the naming format of [YYYY-MM-DD-HHMM_ClimateChamber_pot_PotNr]. The Excel-workbook contains all data collected within the experiment, including its own ReadMe-sheet reporting necessary meta data. Within the Excel-workbook also the processed photosynthesis raw data (all single files bound together to one) as well as the derived ACi parameters. Thus, there is no need to process raw data and recalculate Aci fit for the users. However, this option is supported within the repository.

The collected experimental data includes:

- Experimental schedule
- Experimental set-up
- Information on used wheat varieties
- Irrigation amounts per pot
- Harvested biomass (fresh, dry and dry matter content)
- Plant developmental information (BBCH & height) as well as leaf water potential
- Climate chamber environmental data
- Photosynthesis data, raw data merged to one long format
- Derived ACi parameters

## 4 Experimental Design, Materials and Methods

### 4.1 Experimental setup

In this experiment, we utilized seeds from 12 common agricultural winter wheat varieties (*Triticum aestivum* L.), which were cultivated under ambient (420 ppm) and elevated (600 ppm) CO_2_ concentrations under field conditions using a Free-Air CO_2_ Enrichment (FACE) experiment throughout the 2022 growing season (see Kottmann et al. [9] for details on the FACE experiment).

The pots used in the experiment measured 10.5 cm in diameter and 40 cm in height. Pots were filled with a 50:50 (volume-%) mixture of LD80 and sand to a bulk density of 1 g cm^−3^. Substrate was filled up to 2 cm under the rim resulting in a total rooting depth of 38 cm and an available rooting volume of 3290.5 cm³. Each pot was sown with three seeds (15th July 2025) in the close center of the pot at a uniform depth of 2 cm. After germination, all but one seedling per pot was removed. Sowing in the center avoids possible root-edge limitations. The pots' narrow design was chosen to complement the wheat varieties' deep-rooting characteristics.

Plants and pots were kept in four climate chambers (160L x 70W x 200H cm) throughout the experiment. Aside from CO_2_ concentration, all environmental conditions within the chambers were kept constant: day conditions were set at 24°C, 67 % relative humidity, and full light at 1000 µmol m^−2^ s⁻¹, while night conditions were maintained at 18°C, 52 % relative humidity, and darkness. These settings result in a moderate and constant vapour pressure deficit (VPD) of 1 kPa during both day and night. Transitions between day and night light conditions were facilitated by a one-hour linear ramp, with sunrise occurring between 5:00 and 6:00 and sunset between 20:00 and 21:00, resulting in a 14-hour day length. Two of the four used chambers were maintained at ambient CO_2_ concentration of 400 ppm and the other two at elevated CO_2_ concentration of 800 ppm. The chambers constantly controlled the environmental settings. Literature agrees that atmospheric CO_2_ values less than 600 ppm are too low to completely saturate carboxylation during photosynthesis [10]. To reduce Rubisco's CO_2_ limitation and maximize the inhibition of competitive oxygenation, we chose an elevated CO_2_ treatment concentration of 800 ppm. This aligns with prior experiments investigating the transgenerational effects of elevated CO_2_ [11].

In total we used 144 pots, representing 12 wheat varieties, 2 maternal CO_2_ conditions, 2 chamber CO_2_ concentrations, and 3 replicates. The 72 pots (12 varieties x 2 maternal concentrations x 3 replicates) per chamber CO_2_ concentrations were randomly split among the two chambers used, so that the 3 replicates per variety were split 2:1 to the available chambers. By this arrangement we avoid possible chamber effects overwriting the actual treatment effects. A stratified block randomization scheme was applied to ensure balanced distribution across the chambers and prevent microclimate biases.

### 4.2 Soil water characteristics and irrigation scheme

The substrate's soil water characteristics were determined using six individual soil samples within the the Hyprop system (UMS, Munich, Germany) to create a water retention curve. The substrate's matric potential at field capacity (Ψ_FC_) corresponds to 0.002357 MPa and a gravimetric water content (θ_FC_) of 30 %. The substrate's permanent wilting point is reflected by a matric potential of 1.4498 MPa (Ψ_PWP_) and a gravimetric water content of 10 % (θ_PWP_). Please note, that the bulk density of the soil in the pots is equal to the density of water, so the volumetric water content mirrors the gravimetric water content. Gravimetric water content (GWC) was calculated as ( (Soil_moist_ - Soil_dry_) / Soil_dry_) * 100. For more details on the determination of soil water characteristics please see Berauer et al. [12].

Pots were irrigated by weight every Monday, Wednesday, and Friday prior to the measurement campaign and daily during the measurement campaign. Pots were irrigated to 30 % GWC (θ_FC_) until August 16th 2024, corresponding to the end of the first measurement campaign (13th–16th August 2024). Pots were randomly assigned to be measured during the first measurement campaign. Afterwards irrigation ceased until pots reached the 20 % GWC corresponding to a soil matric potential of 0.012 MPa. This specific GWC value was determined to represent Ψ_csoil_ (*sensu* Cai et al. [2]), indicating the point at which soil conductivity falls below root hydraulic conductivity, marking the onset of soil limitations on water flux within the soil-plant-atmosphere continuum (SPAC) [12]. During the second measurement campaign (21st–23rd of August 2024) each individual plant was measured after it reached and was kept by irrigation at the target level of 20 % GWC, corresponding to Ψ_csoil_. This approach aligns with the recommendations of Knipfer et al. [13], who suggest that a period of three full days under the specified environmental conditions is sufficient to achieve equilibrium within the SPAC while avoiding morphological adaptations to drought stress, such as root suberization or changes in vessel diameters.

### 4.3 Plant ecological measurements

At each of the two measurement campaigns for each individual, we first measured an Aci-curve using the Dynamic Assimilation Technique (DAT) following Saathoff & Welles [14], followed by measuring leaf water potential (Ψ_leaf_), BBCH and total plant height.

Technical, computational and theoretical progress in recent years allowed to quantify photosynthesis response curves in unsteady states [[14], [15], [16]]. This technique is – similar to traditional steady-state measurements – based on the mass balance of the leaf cuvette, but we now can acquire response curves faster with a higher data density. Consequently, we can increase the amount of measured samples in a given time without losing accuracy on parameter estimates, i.e. maximum Rubisco carboxylation rate [Vc_max_] and maximum rate of electron transport [J_max_] [14,16].

We measured the ACi curves on the youngest fully developed leaf of the main stem on each individual, ensuring physiological comparability. Measurements were started three hours after the lights had fully turned on to ensure plant photosynthetic activity. For the measurements, we used two portable gas exchange systems (Li-6800, LiCor, USA), with similar settings. Pots were randomly assigned to one of the two instruments to not introduce a possible instrument effect. On each day we measured 24 pots per instrument. We used as settings saturating light conditions of 1500 µmol m^−2^ s⁻¹, air temperature of 24°C, VPD leaf of 1.5 kPa, flow rate of 600 µmol s⁻¹ with an underpressure of 0.2 kPa, ventilator speed of 10000 rpm. We ramped CO_2_ concentrations from 10 ppm to 1210 ppm with a ramping rate of 100 ppm per minute. Prior to the start of each ramp, we used a 4-minute burn-in period at 10 ppm. Data was automatically recorded every 5 seconds as the 5-second average. Necessary range match (CO_2_ and H_2_O) and dynamic tuning were done twice a day, once before the first plant was measured and once after finishing half of the daily amount. Our approach follows best practice recommendations [16,17].

Leaf water potential (Ψ_leaf_) was measured using a Scholander pressure bomb (Model 1000, PMS Instrument Company, USA), following the procedure outlined by Rodriguez-Dominguez et al. [18]. Ψ_leaf_ was measured on the youngest fully developed leaf of the first tiller (not the main stem) immediately after the individual's photosynthesis was measured. Further, BBCH and total plant height were recorded.

The measurement procedure was similar during both measurement campaigns to ensure consistency and comparability across time points.

After finishing the second measurement campaign on the 23rd of August, plants were harvested 2 cm aboveground. Fresh weight was immediately determined and dry weight after drying to constant weight in an oven at 60°C for a minimum of 48 hours.

### 4.4 Calculation of photosynthetic efficiency (ACi curve fitting)

We estimated maximum Rubisco carboxylation rate [Vc_max_], maximum rate of electron transport [J_max_] and dark respiration [Rd] from ACi curves (based on the model of Farquhar et al. [19] fitted to the measured assimilation rates using the plantecophys-R-package (v.1.4-6 [20]), with the “default” fitting method and without fitting TPU limitation.

## Limitations

Photosynthesis of four replicates was measured only once. See Table 1 for replicate ID and measurement campaign.

**Table 1:** Gives information when pots were measured, if they were not measured during both measurement campaigns.

Replicate ID (pot_nr)	Well Watered conditions	Limiting Watered conditions
26	1	0
50	0	1
135	0	1
142	0	1

## Ethics Statement

We ensure that our study was conducted in full compliance with ethical guidelines. No harm was caused to plants, animals, or humans. No data was sourced from social media platforms. All authors confirm adherence to ethical standards required for publication in Data in Brief.

## CRediT authorship contribution statement

**Bernd J. Berauer:** Conceptualization, Methodology, Software, Data curation, Writing – original draft. **Suraj Chaudhary:** Methodology, Data curation. **Lorenz Kottmann:** Writing – review & editing, Resources. **Andreas H. Schweiger:** Conceptualization, Writing – review & editing.

## Data Availability

ZenodoData on transgenerational memory effects of photosynthetic efficiency of twelve wheat varieties under elevated carbon dioxide concentration and reduced soil water availability (Original data).

## References

[1] Berauer B. (2025). Data & Analysis script for: data on transgenerational memory effects of photosynthetic efficiency of twelve wheat varieties under elevated carbon dioxide concentration and reduced soil water availability. Zenodo.

[2] G. Cai, | Mutez, A. Ahmed, M. Abdalla, and A. Carminati, “Root hydraulic phenotypes impacting water uptake in drying soils,” 2022, doi: 10.1111/pce.14259. 10.1111/pce.14259 PMC9303794 35037263

[3] Trenberth K.E. (2013). Global warming and changes in drought. Nat. Clim. Change.

[4] Böhnisch A., Mittermeier M., Leduc M., Ludwig R. (2021). Hot spots and climate trends of meteorological droughts in europe–assessing the percent of normal index in a single-model initial-condition large ensemble. Front. Water.

[5] McCaw B.A., Stevenson T.J., Lancaster L.T. (2020). Epigenetic responses to temperature and climate. Integr. Comp. Biol..

[6] Kaur V., Singh S., Behl R.K. (2016). Heat and drought tolerance in wheat: integration of physiological and genetic platforms for better performance under stress. Ekin J. Crop Breed. Genet..

[7] Bowes G. (1993). Facing the inevitable: plants and increasing atmospheric CO2. Annu. Rev. Plant Physiol. Plant Mol. Biol..

[8] Walter J., Harter D.E.V., Beierkuhnlein C., Jentsch A. (2016). Transgenerational effects of extreme weather: perennial plant offspring show modified germination, growth and stoichiometry. J. Ecol..

[9] Kottmann L., Kretschmer L., Carotenuto F., Zaldei A., Brilli L. (2025). Experimental design and performance of a free air carbon dioxide enrichment facility in Northern Germany. J. Agron. Crop Sci..

[10] Nowak R.S., Ellsworth D.S., Smith S.D. (May 2004). Functional responses of plants to elevated atmospheric CO2– do photosynthetic and productivity data from FACE experiments support early predictions?. New Phytologist.

[11] Li Y., Li X., Yu J., Liu F. (2017). Effect of the transgenerational exposure to elevated CO2 on the drought response of winter wheat: stomatal control and water use efficiency. Environ. Exp. Bot..

[12] B. J. Berauer, A. Steppuhn, and A. H. Schweiger, “The multidimensionality of plant drought stress: The relative importance of edaphic and atmospheric drought,” 2024, doi: 10.1111/pce.15012. 10.1111/pce.15012 38940730

[13] Knipfer T. (2020). Predicting stomatal closure and turgor loss in woody plants using predawn and midday water potential. Plant Physiol..

[14] Saathoff A.J., Welles J. (2021). Gas exchange measurements in the unsteady state. Plant Cell Environ..

[15] Stinziano J.R., Morgan P.B., Lynch D.J., Saathoff A.J., McDermitt D.K., Hanson D.T. (2017). The rapid A–Ci response: photosynthesis in the phenomic era. Plant Cell Environ..

[16] Stinziano J.R., McDermitt D.K., Lynch D.J., Saathoff A.J., Morgan P.B., Hanson D.T. (2019).

[17] Tejera-Nieves M., Gregory L.M., Saathoff A., Walker B.J. (2024).

[18] C. M. Rodriguez-Dominguez et al., “Leaf water potential measurements using the pressure chamber: synthetic testing of assumptions towards best practices for precision and accuracy,” 2022, doi: 10.1111/pce.14330. 10.1111/pce.14330 PMC9322401 35394651

[19] Farquhar G.D., von Caemmerer S., Berry J.A. (1980). A biochemical model of photosynthetic CO2 assimilation in leaves of C3 species. Planta.

[20] Duursma R.A. (2015).

